# Age-Related Changes of the Anticipatory Postural Adjustments During Gait Initiation Preceded by Vibration of Lower Leg Muscles

**DOI:** 10.3389/fnhum.2021.771446

**Published:** 2021-10-21

**Authors:** Jana Kimijanová, Diana Bzdúšková, Zuzana Hirjaková, František Hlavačka

**Affiliations:** Department of Behavioral Neuroscience, Institute of Normal and Pathological Physiology, Centre of Experimental Medicine, Slovak Academy of Sciences, Bratislava, Slovakia

**Keywords:** gait initiation, anticipatory postural adjustments, age, proprioception, muscle vibration

## Abstract

Gait initiation (GI) challenges the balance control system, especially in the elderly. To date, however, there is no consensus about the age effect on the anticipatory postural adjustments (APAs). There is also a lack of research on APAs in older adults after proprioceptive perturbation in the sagittal plane. This study aimed to compare the ability of young and older participants to generate APAs in response to the vibratory-induced perturbation delivered immediately before GI. Twenty-two young and 22 older adults performed a series of GI trials: (1) without previous vibration; (2) preceded by the vibration of triceps surae muscles; and (3) preceded by the vibration of tibialis anterior muscles. The APAs magnitude, velocity, time-to-peak, and duration were extracted from the center of pressure displacement in the sagittal plane. Young participants significantly modified their APAs during GI, whereas older adults did not markedly change their APAs when the body vertical was shifted neither backward nor forward. Significant age-related declines in APAs were observed also regardless of the altered proprioception.The results show that young adults actively responded to the altered proprioception from lower leg muscles and sensitively scaled APAs according to the actual position of the body verticality. Contrary, older adults were unable to adjust their postural responses indicating that the challenging transition from standing to walking probably requires higher reliance on the visual input. The understanding of age-related differences in APAs may help to design training programs for the elderly specifically targeted to improve balance control in different sensory conditions, particularly during gait initiation.

## Introduction

One of the most important requirements of successful locomotion is the ability to adapt gait to the various demands of the environment (Shumway-Cook and Woollacott, 2007). Motor adaptation, playing a significant role, can be defined as the process of making feedforward modifications or adjustments based on sensorimotor integration to already well-learned motor skills (Martin et al., 1996). The transition from standing to walking, i.e., gait initiation (GI) is known to be a highly challenging task for the balance control system involving correct sequencing of movement preparation and execution. Immediately prior to the stepping, anticipatory postural adjustments (APAs) act to accelerate the center of body mass (CoM) forward and towards the stance leg by moving the center of pressure (CoP) backward and towards the stepping leg in order to minimize the potential imbalance at the moment of single-leg stance (Crenna and Frigo, 1991; Jian et al., 1993). APAs along the anterior-posterior (AP) axis are predictive of motor performance (Lepers and Brenière, 1995) while APAs along the medio-lateral (ML) axis are predictive of postural stability (McIlroy and Maki, 1999; Honeine et al., 2016). In addition, as older individuals are more likely to fall during common daily activities, such as walking, changing position (Tinetti et al., 1995), or initiating a step in different environments (Polcyn et al., 1998; Henriksson and Hirschfeld, 2005), it is particularly important to appropriately respond to either externally or internally generated perturbation stimuli and produce corresponding balance adjustments during these locomotor tasks. Regarding this, GI provides an ideal task to investigate age-related changes in APAs, especially when a sudden change in sensory signals occurs. During the unperturbed stepping, previous studies reported characteristic modifications of APAs in older adults, for instance, reduced initial backward CoP shift, reduced velocity of CoM at the time of first swing foot toe off (Halliday et al., 1998; Khanmohammadi et al., 2015), or significantly diminished momentum-generating capacity of CoP shift (Polcyn et al., 1998). Due to aging, a delayed onset activity of tibialis anterior (Khanmohammadi et al., 2015), more variable muscle activity (Mickelborough et al., 2004), or even lack of anticipatory activity of lower leg muscles (Henriksson and Hirschfeld, 2005) were shown. The older adults exhibited a longer unloading phase with a higher frequency of ground reaction forces peaks and an earlier maximal force rate achieved during this phase (Jonsson et al., 2007). However, other studies reported no age effect (Patchay et al., 2002; Plate et al., 2016; Lu et al., 2017) or only a partial effect of aging (Jonsson et al., 2007) on APAs. Hence, generally, there is no consensus about the age effect on APAs during GI. Moreover, despite the fact that imbalance or falls frequently occur while moving the body in the sagittal plane and position of the body verticality is compromised currently in this plane (Robinovitch et al., 2013), previous studies have investigated APAs mostly in terms of lateral weight shift. Furthermore, to our knowledge, there is a complete lack of research on APAs in older adults when the proprioceptive system was perturbed in the sagittal plane before or during GI. If there are reports which elaborate about the changes in APAs associated with balance perturbation, they have examined postural responses in the frontal plane and mostly in young subjects (Ruget et al., 2008, 2010; Mouchnino and Blouin, 2013; Mille et al., 2014). These studies indicated that young adults are able to modify their APAs when the body is subjected to an external perturbation. If this is true also for older adults, who are more vulnerable to fall in the occurrence of balance perturbation in the sagittal plane, is still unclear. There is a general agreement that proprioceptive signals from leg muscles provide the primary source of information for postural control (see review by Henry and Baudry, 2019). However, aging is associated with a decrease in sensitivity, acuity, and integration of the proprioceptive signal resulting in reduced efficiency of postural control (Deshpande et al., 2016). Therefore, we assume that the age-related impairments in detection and processing of the proprioceptive signals would influence the postural adjustments of GI preceded by the vibration of lower leg muscles.

In the present study, we dealt with this issue by comparing the ability of young and older adults to generate the appropriate APAs in response to the vibratory-induced perturbation delivered immediately before GI. To our knowledge, no study to date examined the age-related differences in APAs due to such perturbation of balance in the sagittal plane. The specific aims were: (1) to determine to what extent the proprioceptive perturbation of balance affects APAs in young and older adults, and (2) to examine whether such an effect is dependent on age. We hypothesized that altered proprioception from lower leg muscles resulting in a modified representation of the body verticality would significantly affect APAs and this would manifest as a change of the APAs magnitude, velocity, and timing in young adults. In contrast, we further hypothesized that older adults would show decreased ability to appropriately respond to the perturbation due to postural and physiological declines associated with aging.

## Materials and Methods

### Participants

Twenty-two young and 22 older adults (Table 1) free of musculoskeletal and neurological disorders that could influence balance control participated in the study. They had no medical history of falls, no pain, numbness, tingling, or weakness at the time of testing. The presence of sensory neuropathy was excluded by Pinprick testing (Nather et al., 2008; Blackmore and Siddiqi, 2016). All subjects gave informed written consent in agreement with the Declaration of Helsinki and the study was approved by the local Ethics Committee.

**Table 1:** Participant characteristics.

	Young (22–35 years)	Older (65–83 years)	*p*-value
*n*	22	22	
Gender (M/F)	9/13	9/13	
Age (years)	29.0 (3.4)	73.7 (5.6)	**<0.001**
Height (m)	1.72 (0.07)	1.63 (0.09)	**<0.001**
Weight (kg)	66.5 (13.3)	73.7 (12.2)	0.08
Foot length (cm)	25.8 (1.7)	25.9 (1.6)	0.73

*Mean (SD). Significant effects are marked in bold*.

### Experimental Setup and Protocol

Gait was initiated from an upright standing on a custom-made force plate (45 × 45 × 6.5 cm; see details in Hirjaková et al., 2017), in-built and located at the beginning of a 5-m walkway. Postural responses were quantified by CoP displacement in the AP direction because changes induced by the vibratory stimulation were also expected in this direction. The CoP data were acquired at 100 Hz sampling frequency and low-pass filtered at 10 Hz cut-off frequency (Mancini et al., 2009; Sinclair et al., 2013). The initial stance position was consistent from trial-to-trial by tracing foot outlines on the force plate.

Before starting each trial, the subjects were required to realign the CoP location to the initial position detected on a monitor. The initial CoP position at vibration onset was arbitrarily assigned a value of zero. Proprioceptive stimulation was delivered by custom-made electromechanical vibrators (DC motor with small eccentric rotating mass, weighing 230 g, cylindrical in shape, 9 cm long with a diameter of 4.7 cm) attached with elastic bands. In the case of triceps surae (TS) muscles, vibrators were fixed to the Achilles tendon of both legs, at the level of the ankle joint, and the vibration-induced body tilt was in the same direction as the forthcoming APAs, i.e., backward. In the case of tibialis anterior (TA) muscles, vibrators were fixed on the tendons of the tibialis anterior, 3–5 cm above the ankle joint, and the vibration-induced body tilt was in the opposite direction as the forthcoming APAs, i.e., forwards. The dimensions and positioning of the vibrators did not disrupt locomotion and they were fixed to the tendons during both vibration and control trials (vibrators off). The activation and deactivation of the vibrators were computer-controlled. The stimulation lasted 5 s with an amplitude of 1 mm and a frequency of 80 Hz. Kinematics of the body (reflective markers placed bilaterally on the following landmarks: acromion, greater trochanter, lateral femoral condyle, lateral malleolus, 5th metatarsal, and heel) was recorded by the optoelectronic motion capture system (BTS Bioengineering, Italy) with a sampling frequency of 100 Hz. The vibration-induced body tilt in the sagittal plane was computed from kinematic data as the angle between the shoulder-hip line, i.e., trunk angle (position of the acromion to hip markers) with respect to the vertical (see details in Abrahámová et al., 2009). The final body tilt induced by vibration was quantified during the last second of vibratory stimulation.

Participants stood on a force plate in a natural upright posture with their arms alongside the body, feet approximately pelvis-width apart, and gaze fixating on a visual target set at the eye level and located 5 m away from the starting stance position. After the initial 5 s of quiet stance followed either by 5 s of vibratory stimulation (vibration trials) or another 5 s of quiet stance (control trials), each participant performed a series of GI trials immediately after hearing an acoustic signal (short “beep”) delivered by the computer. Participants were instructed to step with their dominant leg at a spontaneous velocity and walk straight ahead to the end of the walkway. The acoustic signal was triggered at the same moment as the vibration offset. Participants performed two practice trials to familiarize with the experimental conditions and then five trials were collected in each of the following conditions (for a total of *N* = 15 trials): (1) GI without previous vibratory stimulation (control trial); (2) GI preceded by tendon vibration of TS muscles; and (3) GI preceded by tendon vibration of TA muscles. The order of conditions was randomized across the subjects.

### Data Processing and Analysis

**Figure 1:**
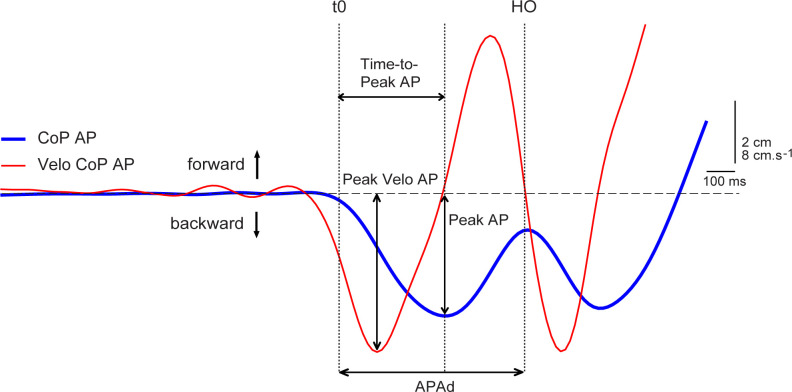
Example of the anticipatory postural adjustments (APAs) variables measured during right-step initiation of the representative young subject during the control trial. Experimental variables evaluated from center of pressure (CoP) displacement (blue line) and velocity of CoP displacement (red line) in anterior-posterior (AP) direction: Peak AP, Time-to-Peak AP, APAd, Peak Velo AP. Vertical dotted lines: t0—onset of APAs; HO—swing limb heel-off.

The APAs variables (Figure 1) were extracted from backward CoP displacement. The onset of APAs was detected by an automated threshold-based algorithm, with the threshold set as 2.5 SD of CoP signal (Caderby et al., 2014) during the initial, pre-vibration period of each trial. The APAs were considered completed (end of APAs) at the time of swing limb heel-off (Rocchi et al., 2006; Delafontaine et al., 2021). The APAs magnitude (*Peak AP*) was measured by the peak of CoP excursion from the CoP position at the moment of vibration offset which was again arbitrarily assigned a value of zero. Related *Time-to-Peak AP*, from the onset of APAs to the instant of Peak AP was also measured (Mancini et al., 2009). The APAs velocity was calculated as a first derivative of CoP displacement and then the peak of CoP velocity (*Peak Velo AP*) was evaluated. The APAs duration (*APAd*) was measured as the time between the onset and the end of APAs (Mancini et al., 2009; Delafontaine et al., 2021). For comparison between the different body dimensions and subjects, CoP displacement was normalized to the foot length and CoP velocity was normalized to height by detrending normalization (O’Malley, 1996) which aims at removing the dependence of stabilometric parameters on anthropometric features (Chiari et al., 2002).

Algorithms for CoP signal analysis and evaluation of the variables were written in MATLAB R2020b (MathWorks Inc., USA). The mean of five trials was used for statistical analyses. Repeated measures ANOVAs were used to test the effect of vibration (TS, C, TA), the effect of age (young, older), and their interaction on each variable after having checked normal distribution with the Shapiro-Wilk test. Greenhouse-Geisser adjustments were performed in cases where the assumption of sphericity was violated. *Post hoc* pairwise comparisons were conducted to identify further differences between TS and C condition, and TA and C condition within each age group. Follow-up independent samples *t*-tests were used on all dependent APAs variables to evaluate the age-related differences. The relationship between the magnitude of vibration-induced body tilt and the APAs magnitude was investigated by a partial correlation analysis while controlling for age. To reduce Type I error due to the multiple *t*-test comparisons, the Bonferroni correction was applied. All statistical analyses were performed using SPSS 18.0 (SPSS, Inc., IL-IBM, USA). The level of significance was set at *p* < 0.05 and effect size (partial eta squared *η*^2^) was also computed.

## Results

**Figure 2:**
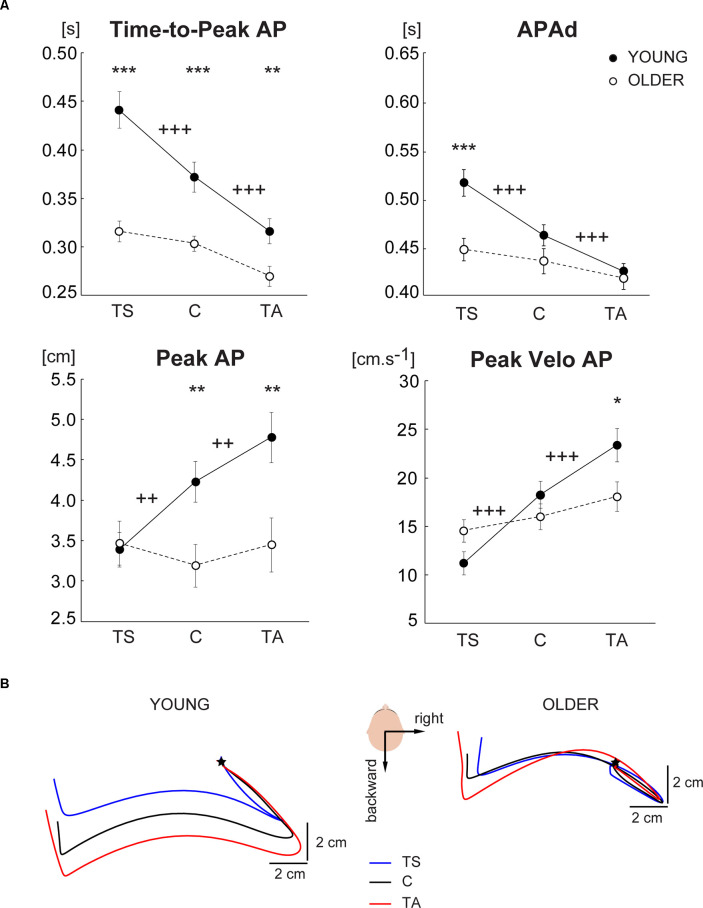
**(A)** Comparison of APAs variables evaluated from CoP displacement in the anterior-posterior direction across three different experimental conditions (TS—vibration of triceps surae muscles, C—control condition with no vibration, TA—vibration of tibialis anterior muscles) in the group of young and older subjects. The values are presented as group mean ± SEM. *Post hoc* differences between vibration conditions and control condition are marked: ^++^*p* < 0.01, ^+++^*p* < 0.001. Differences between young and older are marked: *p < 0.05, **p < 0.01, ***p < 0.001. **(B)** A bird’s-eye view of the mean CoP trajectories during right-step initiation of representative young (left) and older (right) subject during tibialis anterior vibration condition (red), triceps surae vibration condition (blue), and control condition (black). The mean CoP trajectories are realigned according to the APAs onset which is represented by the star.

Significant main effects of vibration, age, and significant effect of vibration by age interaction were observed on all evaluated APAs variables (Table 2), except Peak Velo AP with no age effect. Further *post hoc* comparisons revealed that in young adults, all APAs variables were significantly different in vibration (TS, TA) compared to control (C) condition (Figure 2A). Young adults demonstrated a significant increase of *Time-to-Peak AP* and *APAd* in TS, and a significant decrease of *Time-to-Peak AP* and *APAd* in TA compared to the control condition. Oppositely, *Peak AP* and *Peak Velo AP* significantly decreased in TS and increased in TA compared to the control condition. Thus, the positional change of the body verticality induced by lower leg muscles vibration immediately before stepping resulted in significantly different APAs in young adults, which is clearly visible also in Figure 2B showing CoP trajectories of one representative young subject during all experimental conditions. Contrary, no significant differences in APAs variables were observed between vibration conditions and control GI in older adults (Figure 2A). Unchanged CoP trajectories of one representative older subject across all experimental conditions are displayed in Figure 2B.

**Table 2:** Summary of repeated measures ANOVAs for all evaluated anticipatory postural adjustments (APAs) variables.

Variable	Effect	*F*	*df*	*p*	$\eta_{\text{p}}^{2}$
APAd	Vibration	34.591	(2, 84)	**<0.001**	0.452
	Age	5.954	(1, 42)	**0.019**	0.124
	Vibration × Age	9.401	(2, 84)	**<0.001**	0.183
					
Time-To-Peak AP	Vibration	33.935	(2, 84)	**<0.001**	0.447
	Age	32.791	(1, 42)	**<0.001**	0.438
	Vibration × Age	7.423	(2, 84)	**0.003**	0.150
					
Peak AP	Vibration	10.965	(2, 84)	**<0.001**	0.207
	Age	5.855	(1, 42)	**0.020**	0.122
	Vibration × Age	12.628	(2, 84)	**<0.001**	0.231
					
Peak Velo AP	Vibration	37.403	(2, 84)	**<0.001**	0.471
	Age	0.144	(1, 42)	0.707	0.003
	Vibration × Age	11.238	(2, 84)	**<0.001**	0.211

*Significant effects are marked in bold. Note: Greenhouse-Geisser corrected values are reported where appropriate*.

The partial correlation analysis showed no significant association between the extent of trunk angle induced by vibration of neither TS (*r* = 0.074, *p* = 0.636), nor TA (*r* = 0.044, *p* = 0.781) muscles and the APAs magnitude. Moreover, young and older participants did not significantly differ neither in backward (0.94 ± 0.19° vs. 0.76 ± 0.10°; *p* = 0.440, respectively), nor in forward (0.98 ± 0.21° vs. 0.70 ± 0.07°, *p* = 0.218, respectively) vibration-induced body tilt.

In addition to different anticipatory postural strategies due to the proprioceptive alteration of balance in young and older adults, further analyses revealed significant age-related declines in the APAs variables regardless of the altered proprioception before GI. The older subjects exhibited a significantly shorter *Time-to-Peak AP* in all experimental conditions compared to young adults (Figure 2A). Interestingly, *APAd* was significantly reduced in the older group only in TS condition. Moreover, a smaller *Peak AP* and *Peak Velo AP* were observed in older adults during control GI as well as GI preceded by TA vibration.

## Discussion

In this study, we compared the ability of young and older individuals to generate the appropriate APAs in response to the balance perturbation delivered immediately before gait initiation by bilateral vibratory stimulation of lower leg muscles. In line with our first hypothesis, the young participants significantly modified their postural adjustments during the preparatory phase of stepping and changed all APAs variables under both vibration conditions. In detail, the TS vibration before stepping resulted in backward body tilt to which young adults responded by smaller APAs magnitude and velocity and prolonged APAs duration and time-to-peak compared to unperturbed stepping. On the other hand, when they initiated a step from forward body tilt induced by the TA vibration, they increased the APAs magnitude and velocity while shortening both temporal parameters. As we expected, APAs were modulated according to the internal representation of the body verticality shifted by the vibration of lower leg muscles either forward or backward suggesting that the initial posture strongly influences forthcoming APAs. Our findings agree with previous studies (Rocchi et al., 2006; Dalton et al., 2011), which demonstrated scaling of APAs according to the initial posture. In line with our results, it was concluded that APAs during GI were modulated according to the proprioceptive information originating from the actual position of the body verticality which was altered by translation of support surface (Burleigh and Horak, 1996; Mouchnino and Blouin, 2013), unstable foam support surface (Chastan et al., 2010), the vibration of ankle muscles (Ruget et al., 2010), or applying resistance force at the pelvis (Mille et al., 2014; Laudani et al., 2021). As suggested in these studies, the central nervous system (CNS) utilizes feed-forward prediction for forthcoming voluntary movement, and utilizes immediate afferent information to modify the centrally initiated postural adjustments associated with stepping. Changes of APAs after the delivery of the proprioceptive perturbation suggest that CNS estimated the potentially destabilizing effects of the altered body verticality and acted to modify postural adjustments of GI in order to ensure the stability of the planned movement. The postural adaptations for GI, with respect to changes in proprioceptive information, support a role for multiple, distributed, and interactive systems for postural control (Burleigh and Horak, 1996). However, we found the APAs modulation according to the actual position of the body verticality only in young adults. Thus, the second hypothesis of the study was met as older adults showed a compromised ability to actively respond to the balance perturbation. Although the older subjects showed a similar trend of changing APAs in terms of increasing/decreasing of variables’ values with respect to the vibration compared to control condition as young adults, these changes were not significant. The first aspect which can play a role is an age-related decrease in the proprioception sensitivity which alters both the structures and the functioning of the proprioceptive system (Henry and Baudry, 2019). Alteration in the muscle spindles and their afferents, along with the integration of the signal at the supraspinal level, have been shown to influence proprioceptive perception and postural control in older adults (Goble et al., 2012). Based on our findings, it can be hypothesized that when older people experience a proprioceptive disturbance while initiating a step, they may not be able to properly respond by adjusting their anticipatory postural strategies. The second aspect includes the role of motor prediction in posture and locomotion coupling (Mille et al., 2014). Our findings may be supported by previous studies (Claudino et al., 2013; Laudani et al., 2021) indicating no postural prediction and movement coupling in older adults. It is possible that older individuals may have difficulties in utilizing APAs due to a lack of the necessary coupling between APAs and the stepping movement (Laudani et al., 2021) and also may have reduced usability and efficacy of APAs to ensure stability following the perturbation (Claudino et al., 2013). Another aspect concerns the age-related differences in the importance of proprioceptive and visual information for maintaining equilibrium. While the muscle proprioception is more important for balance control than vision in healthy young adults (Eysel-Gosepath et al., 2016), a decreased effect of proprioceptive alteration with aging indicated a lesser reliance on leg proprioception in older adults (Penzer et al., 2015; Deshpande et al., 2016). In line with this, the declines in peripheral sensory perception with aging caused elevated reliance on visual feedback (Franz et al., 2015).

In the current study, we further aimed to investigate the age-related differences in APAs regardless of proprioceptive alteration. During the unperturbed stepping, older adults showed significantly smaller APAs magnitude and shorter Time-to-Peak AP compared to young adults. Congruently, age-related changes in APAs were revealed in previous studies (Henriksson and Hirschfeld, 2005; Jonsson et al., 2007; Khanmohammadi et al., 2015; Laudani et al., 2021), but there are also studies which reported APAs unaffected by age (Plate et al., 2016; Lu et al., 2017). The findings of Plate et al. (2016), however, do not quite reflect the age-related changes in terms of young vs. old, as they considered as young the adults with mean age 43.8 ± 2.9 years and compared them with middle-aged and older adults (mean age 55.3 ± 2.8 and 66.4 ± 4.5 years, respectively). In the same line, Lu et al. (2017) reported an unchanged spatiotemporal profile of the APAs with aging (ages 20–79). Incongruence with our results may come from different instructions for gait initiation, i.e., self-initiated stepping.

Apart from a significantly reduced APAs magnitude and Time-to-Peak AP due to age in control GI, we found similarly reduced APAs variables also in TA vibration condition. However, in the TS vibration condition, older subjects showed a very similar magnitude of APAs as young adults, but the postural adjustments were significantly shorter and slightly faster than in young adults. We speculate that these different anticipatory adjustments in older adults between the two vibration conditions might be associated with a specific response to the vibration offset (i.e., the moment of vibratory stimulation turn off; Bzdúšková et al., 2018). Specifically, we assume that two autonomic motor control programs, i.e., response to vibration offset and forthcoming APAs came into conflict at the moment when the vibration suddenly stopped and simultaneously the acoustic signal indicated to initiate a step. The postural response to the vibration offset in case of soleus muscles vibration is characterized by a backward shift of CoP displacement followed by a forward CoP overshoot in order to establish equilibrium after the perturbation (Bzdúšková et al., 2018). Oppositely, the response to the TA vibration offset is performed as a forward body tilt followed by a backward CoP overshoot. Therefore, as APAs during GI are known as backward CoP shift, overshoot after the TA vibration offset occurs in the direction of forthcoming APAs and contrary, overshoot after the TS vibration offset occurs in the opposite direction as forthcoming APAs. Our results suggest that the latter case might be more challenging for older adults and indicate higher reliance on vision and adopting a stiffening strategy to prevent the loss of balance. In particular, when older adults experienced a significantly larger and faster overshoot after the TS vibration offset (Bzdúšková et al., 2018) and concurrently they had to initiate a step they might have less time for appropriate APAs resulting in faster and thus shorter APAs compared to young adults.

Interestingly, we revealed a significant decrease due to age in parameter APAd only in the TS condition, while Time-to-Peak AP was significantly different between the age groups in all experimental conditions. This indicates that Time-to-Peak would be a more sensitive parameter to detect early APAs impairments as total APAs duration.

A strength of the study was the comparison of the ability to generate APAs in response to the proprioceptive perturbation of balance both forward and backward. For the first time, hence, this study uncovered and reported sensitive scaling of APAs in the sagittal plane according to the externally shifted vertical in young adults, and revealed the inability to modify APAs in older adults. Therefore, these results should be taken into account when designing training and rehabilitation programs for the elderly specifically targeted to improve balance control in different sensory conditions, particularly during the transition from standing to walking in order to enhance a more accurate adjusting of APAs and potentially prevent falling. On the other hand, we acknowledge several limitations of the present study. First, we did not collect electromyographic signals from lower leg muscles during the vibratory stimulation and GI which could provide additional information about APAs and would help to interpret age-related differences. Second, we might benefit from the kinematic analysis of the first step performed immediately after the balance perturbation. Further studies should address these limitations as well as investigate whether it is possible to improve the proprioception from lower leg muscles and thus increase the anticipatory responsiveness to perturbation.

In summary, aging led to significantly reduced APAs during GI performed from the vibratory shifted vertical as well as from the natural body verticality. Moreover, young adults markedly modulated APAs in response to the vibration delivered before GI, which was in contrast with older adults who showed diminished modulation of APAs. Therefore we can conclude that the ability to generate the appropriate APAs in response to vibratory-induced perturbation particularly in the sagittal plane is age-dependent.

## Data Availability Statement

The raw data supporting the conclusions of this article will be made available by the authors, without undue reservation.

## Ethics Statement

The studies involving human participants were reviewed and approved by the local Ethics Committee of the Center of Experimental Medicine of the Slovak Academy of Sciences. The patients/participants provided their written informed consent to participate in this study.

## Author Contributions

JK, ZH, and FH contributed to the conception and design of the study. JK organized the database and wrote the first draft of the manuscript. JK and DB performed the statistical analysis. JK, DB, ZH, and FH reviewed the manuscript. All authors contributed to the article and approved the submitted version.

## Conflict of Interest

The authors declare that the research was conducted in the absence of any commercial or financial relationships that could be construed as a potential conflict of interest.

## Publisher’s Note

All claims expressed in this article are solely those of the authors and do not necessarily represent those of their affiliated organizations, or those of the publisher, the editors and the reviewers. Any product that may be evaluated in this article, or claim that may be made by its manufacturer, is not guaranteed or endorsed by the publisher.
